# Structural insights into zinc oxide–silver nanocomposite *via* different XRD models: rapid synthesis with photocatalytic & antibacterial applications

**DOI:** 10.1039/d5na00790a

**Published:** 2025-11-03

**Authors:** Md. Abdus Samad Azad, Md. Shahadat Hossain, Shassatha Paul Saikat, Md. Rifat Hasan, Shukanta Bhowmik

**Affiliations:** a Department of Applied Chemistry and Chemical Engineering, Noakhali Science and Technology University Noakhali-3814 Bangladesh shukantabhowmik@nstu.edu.bd; b Department of Chemistry, Noakhali Science and Technology University Noakhali-3814 Bangladesh

## Abstract

In this work, a silver-coated zinc oxide nanocomposite (ZnO/Ag NC) was synthesized *via* a sol–gel method combined with ultrasonic irradiation. SEM analysis revealed uniform particle size and morphology, while UV-vis spectroscopy showed a slight absorption shift due to silver incorporation, and FT-IR confirmed successful Ag coating on ZnO nanoparticles, which was further applied to degrade methylene blue dye and showed stronger antibacterial activity against both Gram-positive and Gram-negative bacteria compared to pure ZnO. Detailed XRD studies confirmed a well-defined wurtzite structure and revealed that the material is semi-crystalline with crystallite sizes ranging from 1 to 100 nm, averaging about 74 nm. Crystallographic parameters such as crystallite size, internal strain, and crystallinity were comprehensively analyzed using multiple models, including linear straight-line, Williamson–Hall, size–strain plot, Halder–Wagner, and Sahadat–Scherrer methods. Among these, the Williamson–Hall method and its variants (uniform deformation, uniform stress deformation, and uniform deformation energy density models) provided the most detailed insights into energy density, lattice stress, and strain. Comparative evaluation of these models confirmed strong correlations in crystallite size estimations, offering a thorough understanding of the nanocomposite's structural properties. These results underscore the promising multifunctionality of the ZnO/Ag NC for environmental and biomedical applications, supported by in-depth crystallographic characterization.

## Abstract 2

In this work, a silver-coated zinc oxide nanocomposite (ZnO/Ag NC) was synthesized *via* a sol–gel method combined with ultrasonic irradiation.

## Graphical abstract

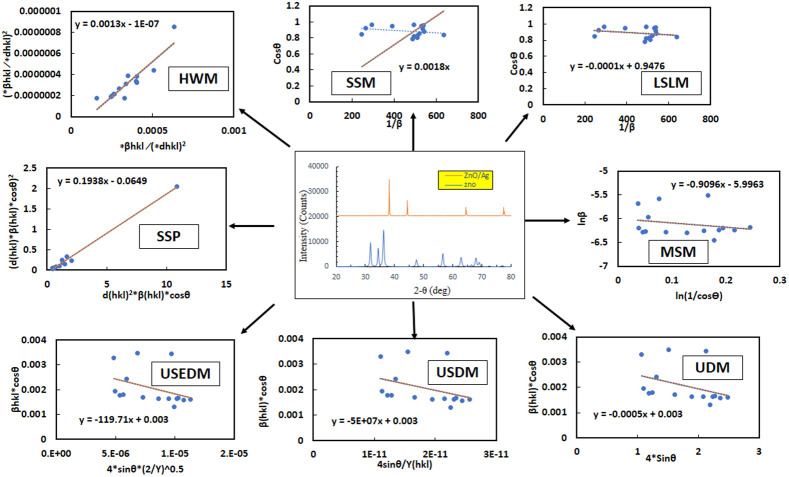

## Introduction

The rapid advancement of nanotechnology has revolutionised materials science, particularly in the development of multifunctional metal oxide nanoparticles (NPs).^1–3^ Among these, zinc oxide (ZnO) NPs have gained remarkable attention due to their wide range of applications spanning photocatalysis,^4^ biosensing,^5^ drug delivery,^6^ environmental remediation,^7^ and antimicrobial activity.^8^ Their widespread utility is attributed to their high surface-area-to-volume ratio,^9^ tunable optical properties,^10^ chemical stability,^11^ and biocompatibility.^12^ ZnO NPs are also recognised for their low toxicity,^13^ making them an ideal candidate for biomedical applications.^14^ Notably, ZnO NPs have demonstrated potential in preserving insulin structure, suggesting their applicability in antidiabetic therapeutics,^15^ in addition to their prominent roles in antibacterial and anticancer treatments.^16,17^ From a structural and electronic standpoint, ZnO stands out among other semiconductors due to its wide direct band gap (∼3.37 eV) and high excitation binding energy (∼60 meV).^18^ These features contribute to its excellent optoelectronic performance and high photocatalytic activity.^19^ ZnO's ability to absorb and reflect ultraviolet (UV) radiation has also made it a frequent choice in self-care products, including sunscreens and cosmetic formulations.^20,21^ However, a significant limitation in the practical application of pure ZnO NPs in photocatalysis lies in the rapid recombination of photoinduced electron–hole pairs, which significantly hampers its efficiency in degrading environmental pollutants or in generating reactive species for antibacterial purposes.^22^

To overcome this, modification of ZnO NPs with noble metals such as silver (Ag) has proven to be an effective strategy.^23,24^ Ag nanoparticles (Ag NPs) exhibit a wide range of beneficial properties, including high surface plasmon resonance,^23^ nonlinear optical behaviour,^25^ and enhanced electron trapping ability.^26^ When incorporated with ZnO, Ag acts as an effective electron sink, reducing the recombination of photo-generated charge carriers and thereby enhancing the formation of reactive oxygen species (ROS) such as hydroxyl radicals (˙OH) and superoxide anions (˙O_2_^−^) under UV or visible light irradiation.^27,28^ Additionally, Ag NPs possess broad-spectrum antimicrobial properties, making the ZnO/Ag NC highly effective in combating both Gram-positive and Gram-negative bacteria.^29^ This synergistic behaviour between ZnO and Ag not only enhances photocatalytic performance but also augments antibacterial efficacy, creating a dual-functional material suitable for environmental and biomedical applications.^27,30^ For instance, Ghosh *et al.* studied stable ZnO/Ag NCs and demonstrated their concentration-dependent antibacterial effects against *E. coli*, *B. thuringiensis*, and *P. aeruginosa*.^8^ On the other hand, Ibanescu *et al.* also explored the synergistic effects of Ag doping on ZnO nanoparticles, highlighting enhanced photocatalytic efficiency and antimicrobial properties;^31^ however, further studies on detailed crystallographic characterization are needed to better understand these enhancements. In addition to electronic effects, the incorporation of Ag into ZnO can lead to crystallographic distortion due to differences in ionic radius and bonding characteristics between Zn^2+^ and Ag^+^ ions.^32^ This distortion can introduce structural defects such as oxygen vacancies or interstitials, which act as additional active sites for ROS generation.^33^ These defects also alter the band structure and optical absorption properties, thereby improving the material's photocatalytic and antimicrobial efficiency.^34^ While effective antimicrobial activity and mechanisms like free radical generation were shown in the literature,^31,35^ there remain gaps in understanding the crystallographic parameters crucial for fully explaining the composite's behavior.^36,37^ In this study, we synthesized ZnO NPs *via* a sol–gel method followed by ultrasonic-assisted Ag coating, aiming to improve photocatalytic and antibacterial performance. But a central part of our work involves extensive characterization of crystallographic parameters using X-ray diffraction (XRD) analysis.

Two main factors influencing XRD peak broadening are lattice strain and crystal size, with intrinsic strain often resulting from grain boundaries and imperfections in the crystalline structure.^38^ Lattice strain mainly arises from dislocations but can also be affected by stacking faults, sinter strains, and grain boundary junctions.^39^ Peak broadening occurs because no crystal is perfect, and the crystallite size (which differs from particle size due to polycrystalline aggregates) largely determines the diffraction peak width. Both size and intrinsic strain affect Bragg peak shapes, and XRD patterns can provide information on crystallinity, crystallite size, lattice parameters, preferred growth, and crystallinity index.^40,41^ While the Scherrer method is widely used to estimate the crystallite size, it does not consider intrinsic strain or instrumental broadening effects.^42,43^

To achieve a more comprehensive understanding of the crystallite size and mechanical properties like lattice strain and stress, several XRD analysis models were applied in this work, including the Linear Straight-Line Method (LSLM), Monshi–Scherrer Method (MSM), Williamson–Hall Method (WHM), Size–Strain Plot (SSP), Halder–Wagner Method (HWM), and Sahadat–Scherrer Method (SSH).^44–46^ Among these, the Williamson–Hall method is highly favored for its simplicity and ability to simultaneously estimate the crystallite size and elastic properties. Its subdivisions – the Uniform Deformation Model (UDM), Uniform Stress Deformation Model (USDM), and Uniform Deformation Energy Density Model (UDEDM) – further allow detailed analysis of anisotropic strain and energy density in the lattice.^47,48^ The SSP method enhances accuracy by modelling XRD peaks as a combination of Lorentzian and Gaussian functions, assigning less weight to less reliable high-angle reflections.^49^ Employing these diverse models enables a thorough comparative investigation of the crystallite size and mechanical characteristics, providing deeper insights into the structural features of the wet-chemically synthesized ZnO/Ag NC based on XRD peak broadening.

## Experimental section

### Chemicals

All reagents used in this study were of analytical grade and obtained from commercial suppliers without any further purification. Zinc acetate dihydrate (≥98%, analytical grade) was procured from Loba Chemie and used as the zinc precursor throughout the experiment. Absolute ethanol (C_2_H_5_OH, 99%) was supplied by Merck, Germany. Silver nitrate (analytical grade), used as the silver precursor for the synthesis of ZnO/Ag NCs, was purchased from Sigma-Aldrich. Sodium hydroxide (NaOH) and sodium borohydride (NaBH_4_) were used as received. Distilled water was used as the solvent for the synthesis of both ZnO NPs and ZnO/Ag NCs.

### Synthesis of ZnO NPs by the sol–gel method

First, 20 g of zinc acetate dihydrate [Zn(CH_3_COO)_2_·2H_2_O] was dissolved in 150 mL of distilled water in a 250 mL beaker and stirred for approximately 20 minutes to prepare the zinc acetate solution. Separately, 80 g of sodium hydroxide (NaOH) was dissolved in 80 mL of distilled water in another 250 mL beaker to form the NaOH solution. The two solutions were then combined in a 500 mL beaker under continuous stirring. Subsequently, 100 mL of absolute ethanol was added dropwise to the mixture with vigorous stirring, and the reaction was allowed to proceed for 90 minutes to obtain a gel-like product. The resulting gel was dried at 80 °C for 12 hours and then calcined in an oven at 250 °C for 4 hours. The final product was obtained as a white powder (Fig. S1).^20^

However, the overall reaction for the preparation of ZnO NPs by using NaOH can be expressed as,Zn(CH_3_COO)_2_·2H_2_O + 2NaOH → ZnO + 2NaCH_3_COO + H_2_O

### Synthesis of the ZnO/Ag NC by the ultrasonic irradiation method

Initially, 0.1 g of the previously prepared ZnO powder was dispersed in 50 mL of distilled water in a 100 mL beaker under continuous stirring. Subsequently, 0.17 g of silver nitrate (AgNO_3_) was added to the dispersion. In a separate 20 mL beaker, 15 mL of 0.02 M sodium borohydride (NaBH_4_) solution was prepared. The NaBH_4_ solution was then added to the ZnO–AgNO_3_ mixture, resulting in the formation of a deep brown-colored solution, indicating the *in situ* reduction of Ag^+^ to metallic Ag nanoparticles (Ag^0^) on the surface of ZnO. This reduction reaction can be represented as:AgNO_3_ + NaBH_4_ + 3H_2_O → Ag^0^ (on ZnO) + NaNO_3_ + B(OH)_3_ + 3.5H_2_

This mixture was subjected to ultrasonic treatment using a probe sonicator for 15 minutes to ensure proper mixing and nanoparticle formation. The resulting product was centrifuged three times at 4000 rpm for 20 minutes each to remove any unreacted components and by-products. The precipitate was washed three times with ethanol to further purify the sample. Finally, the product was dried at 80 °C for 2 hours in a vacuum oven, followed by calcination at 500 °C for 1 hour. The resulting nanocomposite contains approximately 48 wt% ZnO and 52 wt% Ag, based on the initial precursors used. The synthesised ZnO/Ag NC (Fig. S2) was then characterised using various advanced analytical techniques (UV-vis, FT-IR, XRD, and SEM) to confirm the formation of the ZnO/Ag NC.

### X-ray diffraction analysis

Phase analysis of the synthesized materials was carried out using a Rigaku Smart Lab X-ray diffractometer. The diffraction data were collected over a 2*θ* range of 10° to 60°, with a step size of 0.01°. Prior to sample analysis, the instrument was calibrated using a standard silicon reference material. The X-ray source was a copper ceramic tube (CuKα radiation, *λ* = 1.54060 Å). To maintain thermal stability of the X-ray tube, a chiller operating at 23 °C with a water flow rate between 4.5 and 4.8 L min^−1^ was employed. X-ray generation was achieved using a current of 50 mA and a voltage of 40 kV, which is standard for copper anodes. Data acquisition followed the Bragg–Brentano para-focusing geometry, and a Ni-Kβ filter was applied to eliminate unwanted Kβ radiation from the signal.

### Photocatalytic performance evaluation

Assessing the photocatalytic efficiency of nanomaterials under light irradiation is critical for understanding their potential in environmental applications, particularly for the degradation of organic pollutants.^50^ In this study, the photocatalytic activities of pure ZnO NPs and the ZnO/Ag NC were investigated using methylene blue (MB) dye as a model pollutant (Fig. S3). The experiments were conducted under visible light to evaluate the degradation behavior of the dye over time. A 40 ppm aqueous solution of MB was prepared using deionised water, and the maximum absorbance of the dye was confirmed at 660 nm using a UV-vis spectrophotometer. The pH of the solution was maintained at neutral. For the photocatalytic tests, 100 mL of the MB solution was placed in a 100 mL beaker, and the catalyst (ZnO NPs or the ZnO/Ag NC) was added at a fixed concentration. The suspension was stirred in the dark for 30 minutes to reach adsorption–desorption equilibrium. Following the dark equilibration period, the beaker was exposed to natural sunlight from 7:00 AM to 5:30 PM. During the photocatalytic reaction, the total solar radiation measured was approximately 5460 Wh m^−2^, with an average irradiance of about 385 W m^−2^. At regular time intervals, 5 mL aliquots were withdrawn from the reaction mixture, filtered to remove catalyst particles, and analysed using a UV-vis spectrophotometer to monitor the change in absorbance at 660 nm corresponding to MB concentration. This experimental setup allowed for the comparative evaluation of the photocatalytic performance of pure ZnO NPs and the ZnO/Ag NC under real sunlight conditions, providing insight into their potential for visible-light-driven dye degradation.

### Antimicrobial activity evaluation

Nanostructured materials like ZnO and the ZnO/Ag NC have garnered considerable attention for their potential use as antimicrobial agents due to their ability to generate reactive oxygen species and disrupt microbial cell membranes.^51,52^ To evaluate their antibacterial efficacy, ZnO NP and ZnO/Ag NC suspensions were tested against representative Gram-positive bacteria (*Staphylococcus aureus* and *Bacillus subtilis*) and a Gram-negative strain (*Shigella flexneri*). The well diffusion method was employed to assess the inhibitory performance of the prepared samples, specifically targeting their capability to rupture bacterial cells and suppress microbial growth.^53^ The nanoparticle suspensions were prepared at a concentration of 5 mg mL^−1^ by dispersing the required amount of ZnO NPs and the ZnO/Ag NC in sterile distilled water, followed by sonication for 15 minutes to ensure uniformity. Antibacterial testing was conducted using *E. coli* DH5α as a reference strain, and ampicillin served as the positive control to benchmark the activity. Fresh bacterial cultures were initiated by transferring morphologically similar colonies from nutrient agar plates into tubes containing 5 mL of Mueller–Hinton broth. After incubation at 37 °C, the bacterial suspensions were standardised to an optical density (OD) between 0.1 and 0.5 using a spectrophotometer. These suspensions were uniformly spread across Mueller–Hinton agar plates using sterile cotton swabs. Wells were then created using a sterile cork borer, and 40 µL of each nanoparticle suspension was added to the designated wells. The plates were incubated at 37 °C for 24 hours. Following incubation, zones of inhibition were measured to determine the antibacterial efficacy of the materials.

## Results and discussion

### UV-vis absorption spectra

**Fig. 1:**
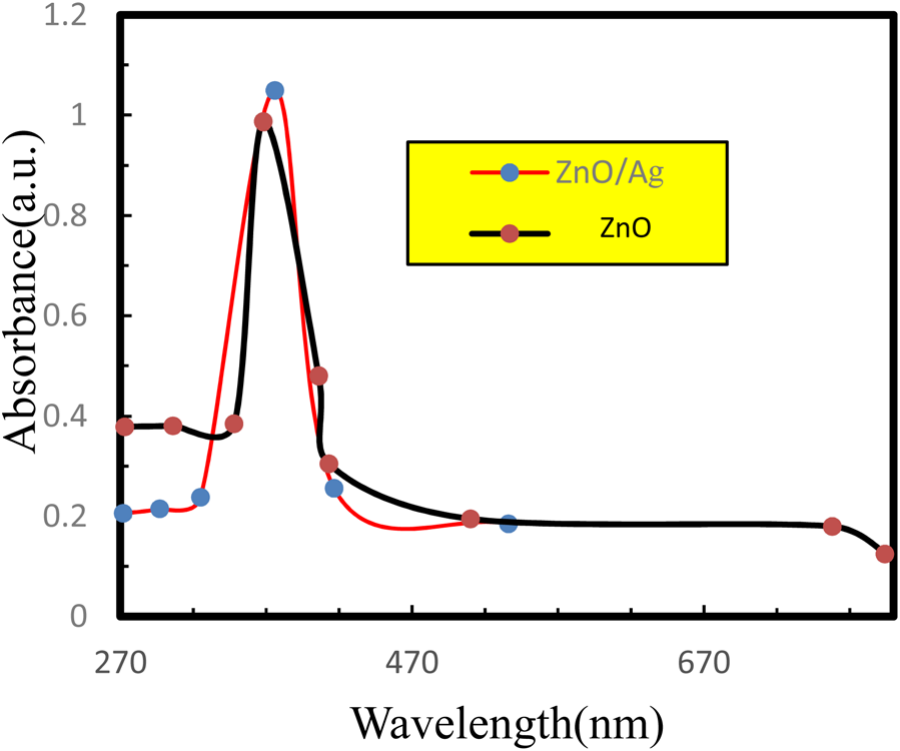
UV-visible spectra of uncoated ZnO NPs and the ZnO/Ag NC.

The UV-vis spectra of the samples were recorded with a SHIMADZU UV-1800 PC UV-vis spectrophotometer over the wavelength range of 200–800 nm. The UV-visible absorption spectra of ZnO NPs and the ZnO/Ag NC are presented in Fig. 1. Uncoated ZnO NPs showed absorption peaks at 368 nm & the ZnO/Ag NC showed a broad peak at 376 nm. It was observed that the absorption maxima of ZnO/Ag was red shifted compared to uncoated ZnO – due to changes in the surface plasmon resonance and local electronic environment caused by Ag incorporation – which confirmed that Ag NPs were coated on ZnO NPs.^31,54,55^

### FT-IR studies

**Fig. 2:**
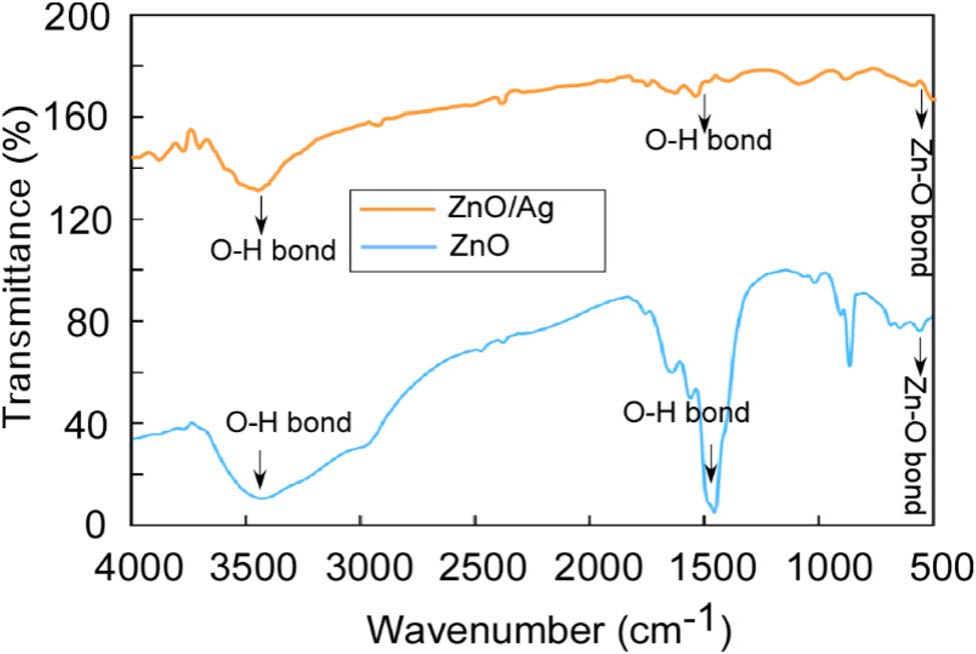
FT-IR spectra of ZnO NPs & the ZnO/Ag NC.

The FT-IR spectrum of ZnO NPs & the ZnO/Ag NC was recorded in the range 400–4000 cm^−1^ and is shown in Fig. 2. From the FT-IR spectrum, various functional groups and metal–oxide (MO) bonds present in the compound were analysed. The vibration band at 500 to 560 cm^−1^ indicates the stretching of the ZnO bond. In the case of the ZnO/Ag NC, the intensity of the peak is reduced due to the incorporation of Ag NPs on the surface of ZnO NPs.^56^ Broad peaks at 3444 cm^−1^ (stretching) and 1450–1750 cm^−1^ (bending) indicate the presence of hydroxyl residue which is due to atmospheric moisture.^57^

### X-ray diffraction (XRD)

**Fig. 3:**
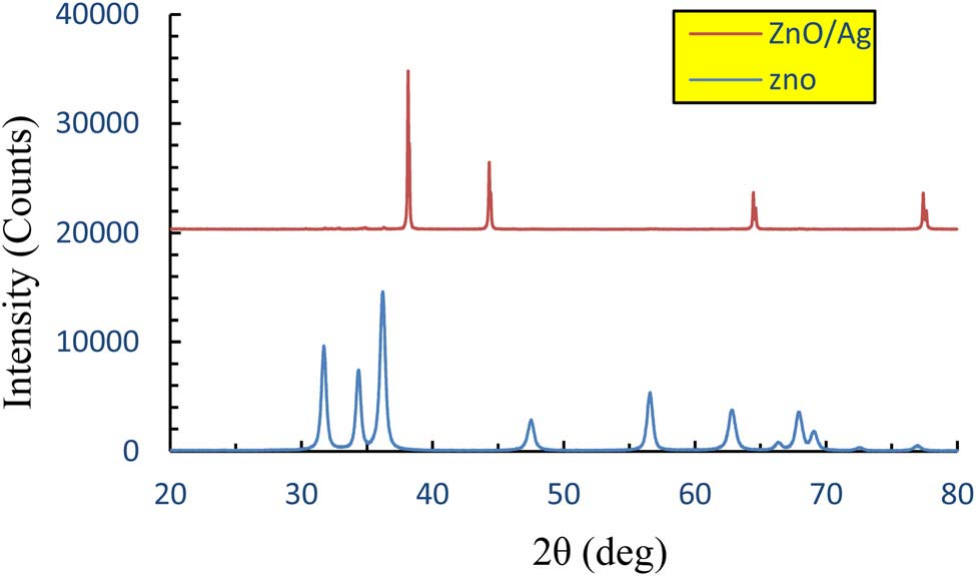
X-ray diffraction (XRD) patterns of ZnO NPs and the ZnO/Ag NC.

XRD patterns of pure ZnO NPs and the synthesized ZnO/Ag NC prepared *via* microwave-assisted synthesis are shown in Fig. 3. A broad diffraction peak centered at 2*θ* = 22.29° appears in both ZnO NPs and ZnO/Ag NC XRD profiles, which is attributed to the ZnO matrix, confirming the successful formation of the ZnO phase under the specified experimental conditions.^56^

Distinct diffraction peaks at 2*θ* values of 37.24°, 43.36°, and 62.86° in the ZnOAg pattern correspond to the (111), (200), and (220) planes of face-centered cubic (fcc) metallic silver, respectively.^58^ These reflections match well with the standard JCPDS card no. 04-0783,^59^ confirming the successful integration of Ag nanoparticles into the ZnO matrix using ethylene glycol (EG) as the reducing agent. The sharpness and intensity of these peaks indicate that the synthesized ZnO/Ag NC possesses high crystallinity.

Additional peaks corresponding to ZnO at 2*θ* = 31.75° (JCPDS card no. 04-0783) and nanoscale Ag at 2*θ* = 44.26° were also observed in Fig. 3.^60^ The absence of any other significant peaks confirms the high phase purity of the synthesized ZnO/Ag NC.^61^

The average crystallite size of the ZnO/Ag NC was calculated to be 73.64 nm using the Scherrer equation (eqn (1)), derived from the FWHM of prominent diffraction peaks:1
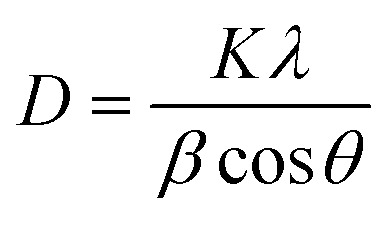


In this equation, *K* is the Scherrer constant (typically 0.9), *λ* is the X-ray wavelength, *β* is the full width at half maximum (in radians), and *θ* is the Bragg angle.

The crystallinity degree (*X*_c_), which represents the volume fraction of the crystalline phase, was determined using:2
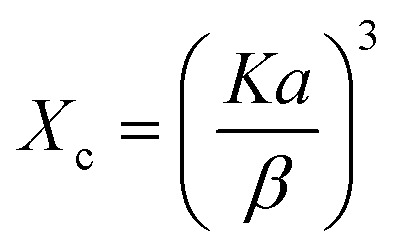


Dislocation density (*δ*), indicating the number of defects in the crystal lattice, was evaluated using the crystallite size through:3
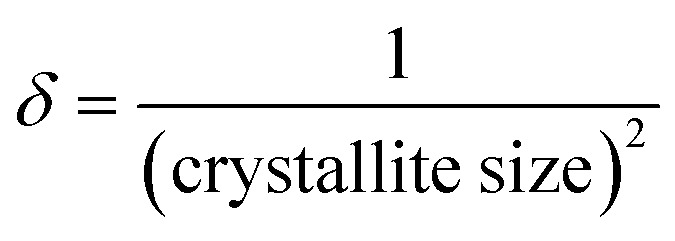


Microstrain (*ε*), which refers to lattice distortion due to local stress or defects, was calculated using:4
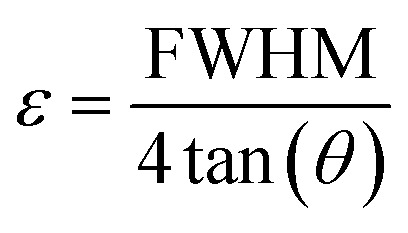
where *D* is the crystallite size, *β* is the FWHM in radians, *θ* is the Bragg angle, *X*_c_ is the crystallinity degree, *δ* is the dislocation density, and *ε* is the microstrain.

In all calculations, the shape factor (*K*) was taken as 0.94 (Table 1). The detailed values of *β* and *θ* used in Scherrer calculations are provided in SI Table S1, and the definition all the symbols used in the models is presented in Table S2 of the SI.

**Table 1:** Different crystallographic results of the synthesized ZnO/Ag NC

Sample	Crystallite size, (nm)	Crystallinity degree	Dislocation density, *δ*	Microstrain, *ε*
Ag-coated ZnO NCs	73.64	8.74	0.0001	−0.0031

### Crystallite characteristics determination using several models

#### LSLM

X-ray diffraction can be used to determine the crystallite size and strain resulting from dislocations by analyzing peak broadening.^62^ The width of a Bragg peak is influenced by both instrumental and sample-related factors.^63^ To separate these effects, a diffraction pattern is recorded for a standard reference material, such as silicon, to quantify the instrumental broadening.^64^ The true broadening of the XRD peak can then be calculated using the following relation:

5 *β*_actual_^2^ = *β*_measured_^2^ − *β*_instrumental_^2^

The Scherrer equation is commonly employed to estimate the average size of crystalline nanoparticles; however, it provides only the minimum crystallite size. The crystallite size is expressed as:6
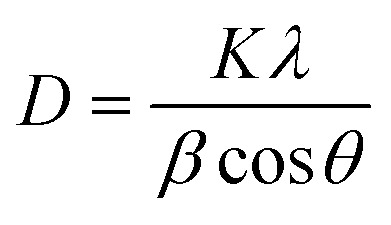
where *D* = crystallite size in nm, *θ* = the measured Bragg angle or diffraction angle, *λ* = wavelength of the radiation (1.54056 Å for CuKα radiation), *K* = shape factor (where *k* = 0.94), and *β* = full-width half maximum intensity (FWHM).

Rearranging eqn (6), we get another relation:7
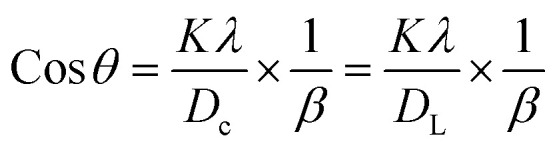


**Fig. 4:**
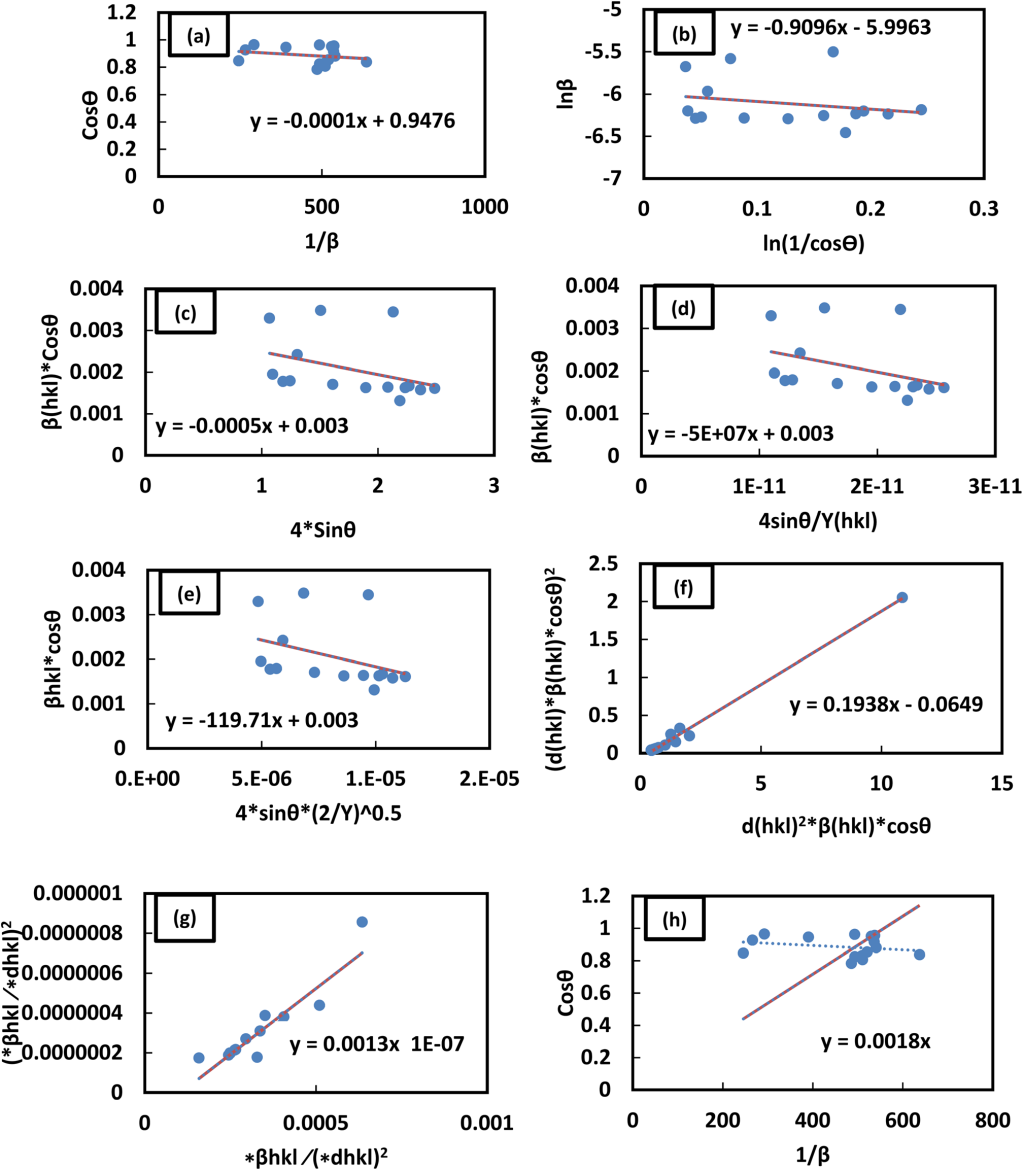
Representation of the (a) LSLM, (b) MSM, (c) UDM, (d) USDM, (e) UDEDM, (f) SSP, (g) HWM, and (h) SSM for ascertaining the crystallite sizes of the synthesized ZnO/Ag NC.

This relation is referred to as Scherrer's linear equation. To determine the crystallite size, a graph is plotted with cos *θ* on the *y*-axis and 1/*β* on the *x*-axis, as shown in Fig. 4(a). The slope of the resulting straight line corresponds to *Kλ*/*D*_L_, from which the value of *D*_L_, representing the crystallite size, can be obtained.

#### MSM (Modified Scherrer Equation)

The Monshi–Scherrer method is widely used to estimate the crystallite size. In materials science, substances are generally categorized as either crystalline or amorphous. X-ray diffraction (XRD) patterns differentiate between these structures: amorphous materials display broad, poorly defined peaks, whereas crystalline materials produce sharp, well-defined peaks. Because XRD is based on diffraction from an ordered crystal lattice, it is suitable only for crystalline materials and is not applicable to amorphous phases.^65^ For a nanocrystalline sample exhibiting N diffraction peaks within the *θ* range of 0°–90° (or equivalently, 2*θ* from 0°–180°), each peak should correspond to the same crystallite size (*D*). A key difficulty in this method, however, is deciding whether the slope obtained from the modified linear regression should be greater or less than unity.^66^ An important advantage of the modified Scherrer method is that it minimizes estimation errors by enabling the use of data from all or selected diffraction peaks, thereby providing a more accurate determination of crystallite size.^67^ The Monshi–Scherrer method is derived from a linearized form of the conventional Scherrer equation.^68^

The general form of the Scherrer equation is:8
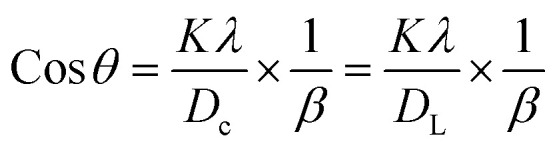
Here, *D*_L_ and *D*_c_ represent the crystalline size obtained using the linear straight line method.^48^

Rearranging the equation, we get9
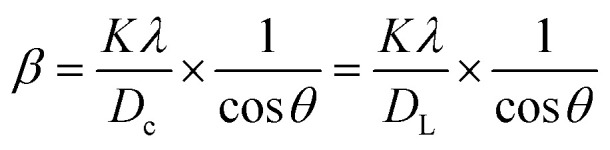


Now, taking ln on both sides of the equation, we can write-10
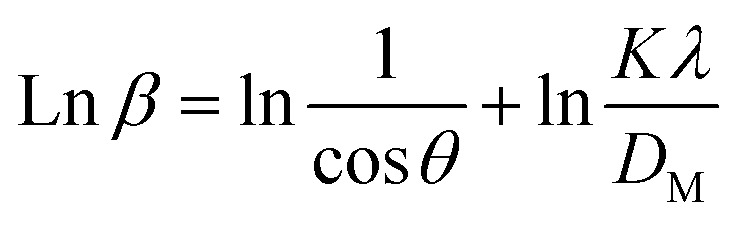


This formula indicates the equation for the Monshi–Scherrer method.

This logarithmic expression forms the basis of the Monshi–Scherrer method. When a plot of 
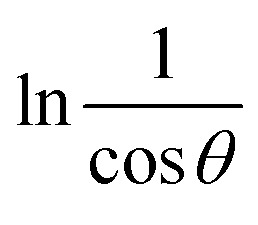
*versus* ln *β* is constructed (as depicted in Fig. 4(b)), the crystallite size can be calculated from the *y*-intercept of the resulting straight line. The crystallite size is then derived from the intercept using the relation:11
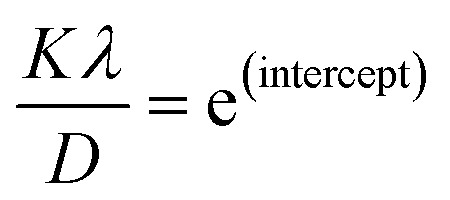


#### WHM

The Scherrer equation assumes ideal, defect-free crystals; however, real crystals often contain imperfections that introduce strain.^69^ To address this, Wilson proposed an equation that estimates strain from peak broadening, with the result influenced by the tan *θ* term.^40,70^ The Williamson–Hall method extends this concept, enabling the simultaneous determination of crystallite size and lattice strain. By incorporating the effects of crystal defects, this approach provides a more accurate characterization of crystalline materials, offering insights into both size and strain that overcome the limitations of the Scherrer equation's idealized assumptions.

In the Williamson–Hall approach, the overall peak broadening of a material, incorporating both size and strain contributions, is expressed using eqn (12).^46^

12 *β*_total_ =*β*_size_ + *β*_strain_

In eqn (12), *β*_strain_ is equal to 4*ε* tan *θ*, and *β*_size_ is equal to *Kλ*/(*D* cos *θ*) (Scherrer's equation). By putting the terms *β*_size_ and *β*_strain_ in eqn (12);13
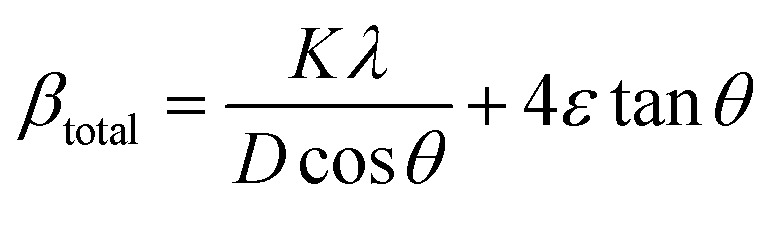
14
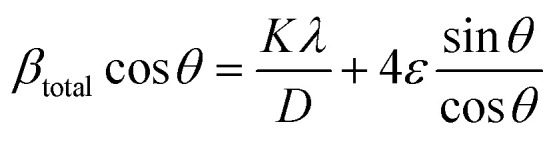


In this case, the strain-broadening effect is represented by *β*_strain_, while the broadening owing to size is defined by *β*_size_. Several sub-analysis methods of the W–H method, including the Uniform Deformation Model (UDM), Uniform Stress Deformation Model (USDM), and Uniform Deformation Energy Density Model (UDEDM), are considered and discussed in this context.

#### UDM

Eqn (15) offers a mathematical approach to quantify the strain in the synthetic ZnO/Ag NC, reflecting the crystal defects and deformations present within the material:^71^15
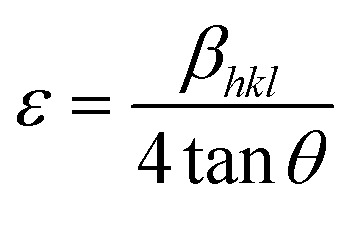


The uniform deformation model assumes that strain is evenly distributed across the entire material, treating it as uniform in all directions regardless of local variations. In XRD patterns, peak broadening is typically attributed to strain within the crystal lattice.

16 *β*_strain_ = 4*ε* sin *θ*

The total broadening *β*_*hkl*_, corresponding to the Full Width at Half Maximum (FWHM) of the reflected peak, results from the combined contributions of crystal lattice strain (*β*_strain_) and crystallite size (*β*_size_) for each peak, as described in eqn (16)–(18).

17 *β*_*hkl*_ = *β*_size_ + *β*_strain_

18
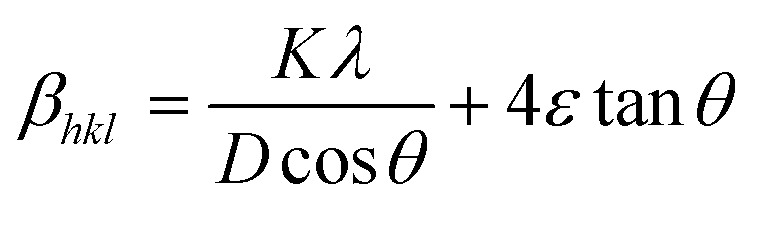


By rearranging eqn (18), eqn (19) is obtained, which is frequently applied within the uniform deformation model framework.^72^19
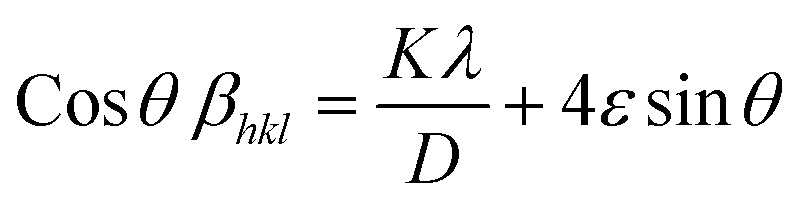


Plotting *β*_(*hkl*)_ × cos *θ* on the *y*-axis against 4 × sin *θ* on the *x*-axis produces a straight line, as shown in Fig. 4(c). The slope of this line corresponds to the strain (*ε*), while the *y*-intercept represents the crystallite size (*D*_W_). These parameters are determined using the linear equation (*y* = *mx* + *c*). The calculated crystallite strain and size derived from this model are summarized in Table 2.

**Table 2:** Crystallographic findings of the synthesized ZnO/Ag NC

Model name		Crystallite size, *D* (nm); strain, *ε* (N m^−2^); energy density, *u* (J m^−3^); stress, *σ*
		ZnO/Ag NC
Linear straight-line model		*D* = 1386 nm
Monshi–Scherrer model		*D* = 55.70 nm
Williamson–Hall model	UDM	*D* = 46.2 nm
		*ε* = −0.0005
	USDM	*D* = 46.2 nm
		*σ* = −4.43 × 10^18^
	UDEDM	*D* = 46.2 nm
		*u* = 5.84 × 10^14^
Size–strain plot		*D* = 76.92 nm
		*ε* = 0.509
Halder–Wagner model		*D* = 5.7 nm
		*ε* = 6.32 × 10^−4^
Sahadat–Scherrer model		*D* = 77 nm

#### USDM

The Uniform Deformation Model (UDM) assumes that the crystal is isotropic and homogeneous; however, this is not always valid. To better reflect real conditions, an anisotropic approach, the Uniform Stress Deformation Model (USDM), was developed as a modification of the Williamson–Hall method.^73^ USDM assumes that the lattice experiences uniform deformation stress in all crystallographic directions, with only a small amount of microstrain present.^69^ This model is suitable when the strain within the nanocrystal is minimal. The relationship between stress and strain is described by Hooke's law, which states that stress and strain are linearly proportional within the elastic limit. Beyond this elastic limit, when strain increases, the uniform stress deviates from linearity.^49^ Mathematically, this is expressed as:

20 *σ* = *εY*_*hkl*_

Here, *σ* represents the crystal's uniform stress, *Y*_*hkl*_ is Young's modulus (modulus of elasticity), and *ε* denotes the strain. By substituting the strain term in eqn (19) with the expression from eqn (20), a new relationship can be derived:21
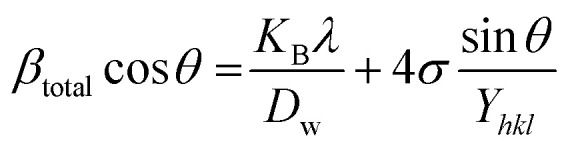


This modified Williamson–Hall equation accounts for uniform stress across all crystallographic directions. By plotting *β*_total_ *cos* *θ* against 
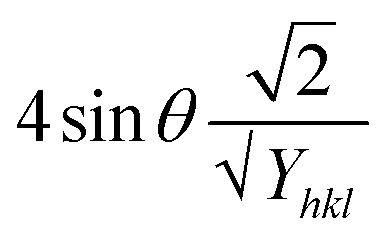
 as shown in Fig. 4(d), the crystallite size and uniform deformation stress can be determined from the *y*-intercept and slope of the linear fit, respectively.

#### UDEDM

While the uniform deformation model assumes crystals are homogeneous and isotropic, many crystals exhibit anisotropy due to lattice deformation. To address this, the Uniform Stress Deformation Model (USDM) was developed. Additionally, in USDM, the stress–strain relationship becomes nonlinear when the crystal's energy density (*u*) is taken into account.^68^ Crystal imperfections arise from various defects, agglomerates, and dislocations. To incorporate these effects, Williamson–Hall proposed an extended method that treats energy density as a function of strain. This model effectively calculates crystallite size (*D*), strain (*ε*), stress (*σ*), and energy density (*u*).^67^ For an elastic system obeying Hooke's law, the energy density (energy per unit volume) can be expressed as:22
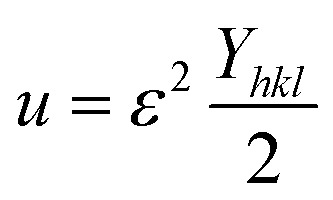


By rearranging the equation, we can write,
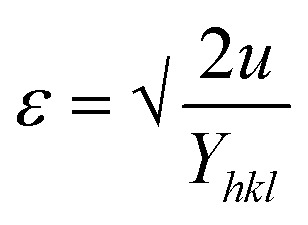


So, eqn (21) can be rewritten based on the energy density and strain relationship:23
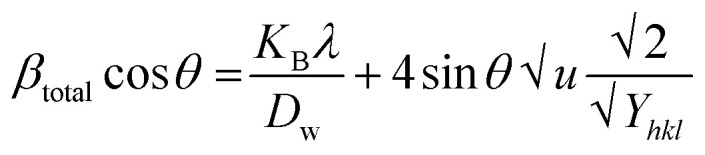


The uniform energy density and crystallite size can be readily determined from the linear plot shown in Fig. 4(e), which graphs *β*_total_ cos *θ* against 
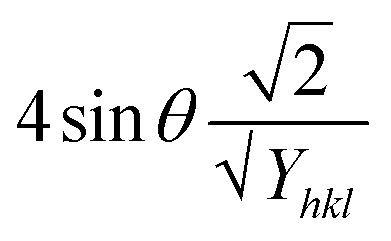
. The *y*-intercept and slope of this line correspond to the uniform energy density and crystallite size, respectively. Additionally, the microstrain of the lattice can be estimated using the value of *Y*_*hkl*_.

#### SSP

The Williamson–Hall plot indicates that peak broadening is isotropic,^68^ implying that microstrain contributes to maintaining isotropy within the diffraction domains. However, a more accurate assessment of size–strain parameters under isotropic broadening can be achieved using the average Size–Strain Plot (SSP). A key benefit of the SSP method is that it assigns less weight to data from high-angle reflections, where measurement accuracy tends to be lower.^47^

This approach utilizes both Gaussian and Lorentzian functions, with the strain profile described by the Gaussian component and the crystallite size characterized by the Lorentzian component.^74^ Accordingly, the total peak broadening can be expressed as a combination of these two functions:

24 *β*_total_ = *β*_L_ + *β*_G_

The peak broadening contributions from the Lorentzian and Gaussian functions are represented by *β*_L_ and *β*_G_, respectively. The Size–Strain Plot (SSP) method also has the advantage of giving greater weight to data collected at low diffraction angles, where measurements are generally more accurate.^46^ The SSP estimation is calculated using the following equation:25
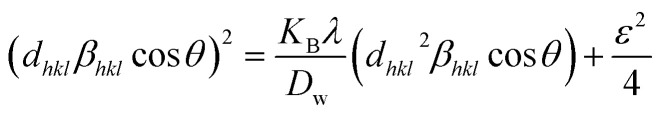


In this context, *ε* denotes the apparent strain, *K* is a shape-dependent constant (with a value of 0.94 for spherical particles), and *d*_*hkl*_ represents the interplanar spacing between the *hkl* lattice planes. Crystallite size and strain can be determined by plotting (*d*_*hkl*_^2^*β*_*hkl*_ cos *θ*) against (*d*_*hkl*_*β*_*hkl*_ cos *θ*)^2^, as shown in Fig. 4(f). The strain is estimated from the square root of the *y*-intercept, while the crystallite size is obtained from the slope of the plot.

#### HWM

In the earlier SSP method, the total XRD peak broadening is attributed to both Gaussian and Lorentzian functions. However, in practical XRD patterns, peak broadening rarely conforms strictly to either function.^46^ The Gaussian function fits the peak well but not its tails, whereas the Lorentzian function matches the tails accurately but not the peak center. To address this limitation, the Halder–Wagner method was introduced, which models peak broadening using a symmetric Voigt function, a convolution of Gaussian and Lorentzian profiles. This approach forms the basis of the method.^75^ According to the Halder–Wagner model, the physical full width at half maximum (FWHM) profiles for the Voigt function can be described as:

26 *β*_*hkl*_^2^ = *β*_L_*β*_*hkl*_ + *β*_G_^2^

In this equation, *β*_L_ and *β*_G_ represent the full width at half maximum (FWHM) contributions from the Lorentzian and Gaussian components, respectively.

Additionally, this method offers the advantage of focusing on peaks at low to medium angles, where there is minimal overlap between diffraction peaks.^76^ Consequently, the relationship between lattice strain and crystallite size can be expressed as follows:27
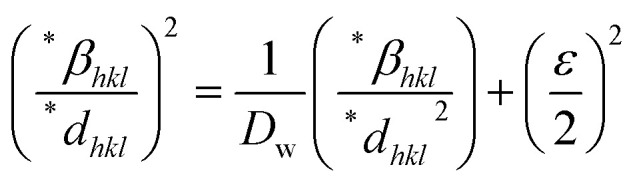
where *β_*hkl*_ = *β*_*hkl*_ cos(*θ*)/*λ*, *d_*hkl*_ = 2*d*_*hkl*_ sin(*θ*)/*λ*.

By plotting 
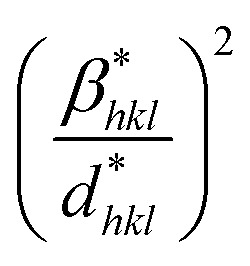
 on the *y*-axis against 
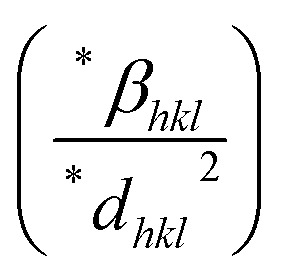
 on the *x*-axis, the crystal strain and crystallite size can be determined from the resulting graph.

The crystallite size is calculated from the *y*-intercept of the straight line shown in Fig. 4(g), while the slope of the line is used to determine the intrinsic strain of the nanocrystal.

#### SSM

Crystalline materials with defined crystallite sizes are crucial for the effective application of nanocrystals. Beyond the Scherrer method, several techniques have been proposed to estimate both intrinsic strain and crystallite size in nanocrystals. Recently, the Sahadat–Scherrer model was refined to address limitations of previous approaches, aiming for more accurate crystallite size determination.^76^ This model considers strain broadening negligible and focuses on removing instrumental broadening effects from the overall peak broadening. Assuming that peak widening is primarily due to crystallite size, the average crystallite size is calculated using an equation constrained to pass through the origin. A linear plot incorporating all reflections is constructed, and the crystallite size is derived from the slope, analogous to the standard linear form (*y* = *mx* + *c*).^46^ Detailed specifications of this model are available in the literature.^73^ The relationship is given by:28
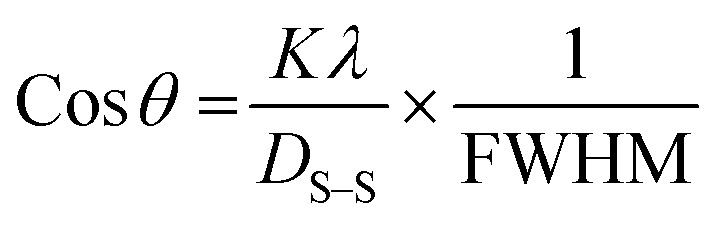


This equation represents the Shahadat–Scherrer model, which offers the advantage of a straightforward calculation of the crystallite size. By plotting cos *θ* on the *x*-axis against 
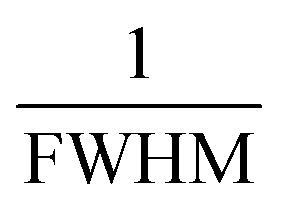
 on the *y*-axis, as shown in Fig. 4(h), a straight line is obtained. The crystallite size is then determined from the slope of this line. The second straight line is used to evaluate the crystalline size by considering the slope 
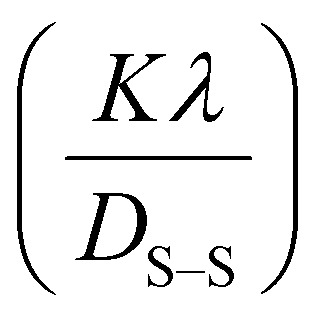
 from eqn (28). Since this line does not intersect the *y*-axis, it yields a more accurate result.^74^

#### Scanning electron microscopy (SEM)

**Fig. 5:**
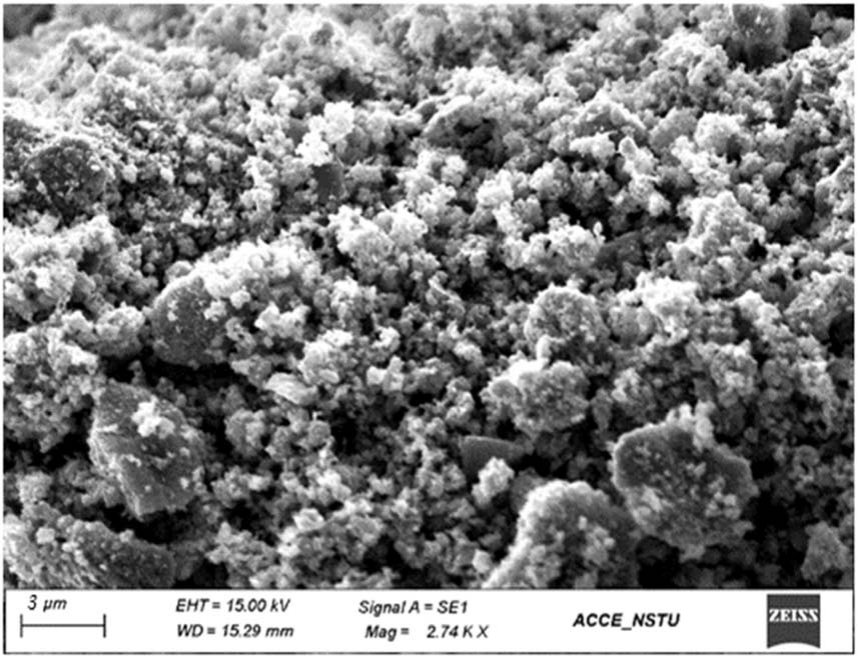
SEM image of the ZnO/Ag NC.

Scanning electron microscopy (SEM) was employed to investigate the morphological characteristics and particle size distribution of the synthesised ZnO NPs and ZnO/Ag NC, which is shown in Fig. 5. The uncoated ZnO NPs exhibited a relatively smooth and uniform surface, whereas the ZnO/Ag NC displayed a rougher texture with distinct Ag NPs uniformly distributed on the ZnO microspheres. This morphological transformation confirms the successful surface modification by Ag NPs. The enhanced surface roughness and heterogeneity are expected to increase the specific surface area, which facilitates greater light absorption and provides more active sites for photocatalytic reactions.^55^ Moreover, the presence of Ag on the ZnO surface is known to inhibit bacterial colonisation and improve reactive oxygen species (ROS) generation under light,^77^ thereby contributing to the enhanced photocatalytic degradation of methylene blue and superior antibacterial performance observed in the study.

#### Evaluation of optical characteristics

**Fig. 6:**
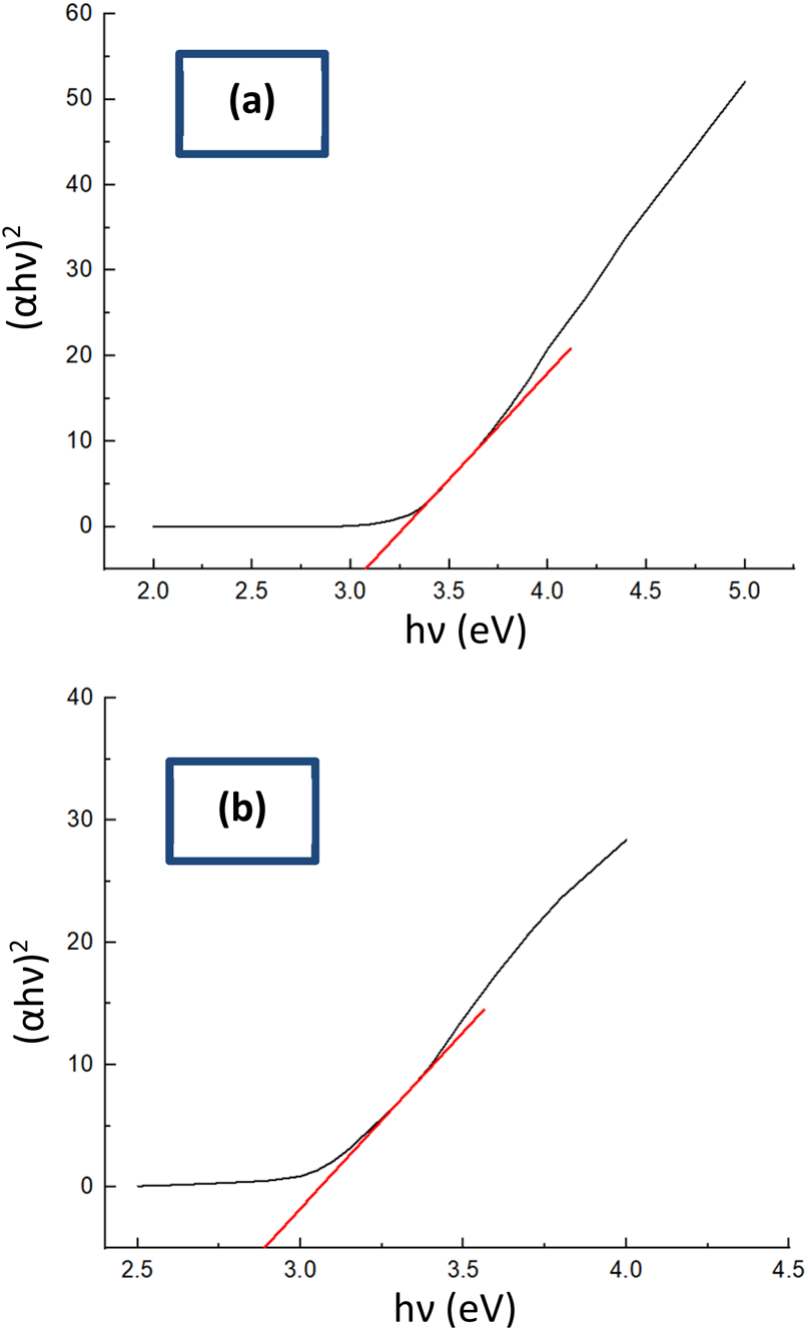
Measured optical bandgap of (a) ZnO NPs, and (b) ZnO/Ag NC from the Tauc plot.

A UV-vis spectrophotometer was employed to determine the optical band gaps (*E*_g_) of ZnO NPs and the ZnO/Ag NC synthesized under controlled conditions at ambient temperature as shown in Table 3. The Tauc plot method was utilized to estimate the absorption edge and corresponding band gap energies, based on the relation:

29 *αhθ* = *A*(*hθ* − *E*_g_)^*n*^

where *α* is the absorption coefficient, *h* is Planck's constant, and *θ* represents the photon frequency mentioned in eqn (29). The obtained results revealed that the pure ZnO NPs exhibited a direct optical band gap of approximately 3.09 eV, consistent with previously reported values for bulk ZnO materials.^78,79^ Upon incorporation of Ag NPs to form the ZnO/Ag NC, a noticeable reduction in the band gap to 2.91 eV was observed (Fig. 6). This narrowing of the band gap is attributed to the interaction between ZnO and Ag, which likely introduces additional electronic states near the conduction band and enhances localized surface plasmon resonance (LSPR) effects.^80^ The presence of Ag NPs may also facilitate charge transfer processes and reduce recombination rates, leading to altered electronic structures and improved optical absorption in the visible region.^77^ These findings suggest that Ag incorporation significantly modifies the optical behavior of ZnO, making the ZnO/Ag NC a promising material for optoelectronic and photocatalytic applications.

**Table 3:** Bandgap value of the synthesized ZnO NPs and ZnO/Ag NC

Sample	*E*_g_ (eV)
ZnO NPs	3.09
ZnO/Ag NCs	2.91

#### Photo-catalytic studies

**Fig. 7:**
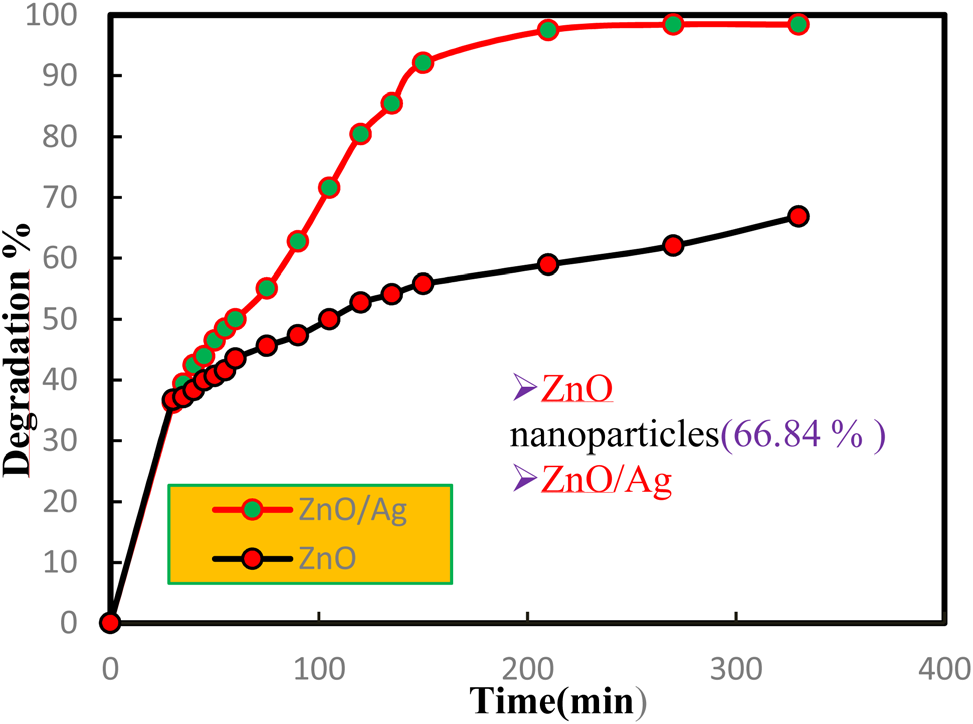
Comparison of the photocatalytic MB dye degradation % curve of ZnO NPs & the ZnO/Ag NC.

Fig. 7 shows the photocatalytic efficiency of pure ZnO NPs and the ZnO/Ag NC by monitoring the change in absorbance of methylene blue (MB) solution at different time intervals under visible light irradiation. The strong absorption band of MB at 660 nm consistently decreased with increasing illumination time. The absorbance of the main MB band dropped from 0.950 to 0.015 after 330 minutes of visible light exposure (preceded by a 30 minutes dark adsorption period) in the presence of the ZnO/Ag NC. In contrast, for pure ZnO NPs, the absorbance decreased from 0.950 to 0.315 under identical conditions. This corresponds to a degradation efficiency of 98.42% for the ZnO/Ag NC compared to 66.84% for pure ZnO NPs.

The significantly enhanced photocatalytic activity of the ZnO/Ag NC can be attributed to several synergistic effects introduced by Ag incorporation. Firstly, Ag NPs act as electron sinks, trapping photogenerated electrons from ZnO and thereby suppressing the recombination of electron–hole pairs.^81^ This prolongs the lifetime of charge carriers and enhances the generation of reactive oxygen species (ROS), which play a critical role in the degradation of organic dyes like MB.^82^ Secondly, the surface plasmon resonance (SPR) effect of Ag NPs under visible light extends the light absorption range of the composite, improving its photocatalytic activity in the visible spectrum.^83^ Lastly, the Ag coating enhances the surface area and modifies the surface morphology (Fig. 5), offering more active sites for photocatalytic reactions. Collectively, these effects contribute to the superior performance of the ZnO/Ag NC in degrading methylene blue under visible light.

It should be noted that the observed 98.42% degradation efficiency is specific to the ZnO/Ag nanocomposite with approximately 48 wt% ZnO and 52 wt% Ag, as prepared in this study. The degradation performance is highly dependent on catalyst composition, dosage, dye concentration, pH, and light intensity.^84^ Therefore, not all ZnO–Ag ratios or loadings will necessarily result in similar degradation efficiencies under identical conditions.

#### Photocatalytic reusability experiments

The reusability or recyclability of a photocatalyst is a critical parameter in assessing its stability, durability, and potential for practical environmental applications.^85,86^ In this study, the photocatalytic performance of ZnO NPs and the ZnO/Ag NC was evaluated over three consecutive degradation cycles of MB dye under visible light irradiation. The experiments were conducted using 0.1 g of catalyst dispersed in 100 mL of MB solution (2.87 × 10^−5^ M), with each cycle lasting 90 minutes. After each cycle, the dye solution was filtered, and the recovered photocatalyst was washed, dried at 60 °C for 2 hours, and reused for the subsequent cycle.

In the first cycle, ZnO NPs demonstrated a degradation efficiency of 66.84%, whereas the ZnO/Ag NC exhibited a significantly higher degradation of 98.42%, highlighting the synergistic effect of Ag incorporation in enhancing visible light activity. During the second cycle, the degradation efficiency slightly decreased to 59.23% for ZnO NPs and 92.15% for the ZnO/Ag NC. This moderate decline can be attributed to the partial occupation of active sites by residual dye molecules and possible surface fouling.^87^ By the third cycle, further reduction in degradation efficiency was observed, with ZnO NPs reaching 51.67% and the ZnO/Ag NC retaining a relatively high value of 87.38%, indicating superior structural stability and catalytic resilience of the nanocomposite.

**Fig. 8:**
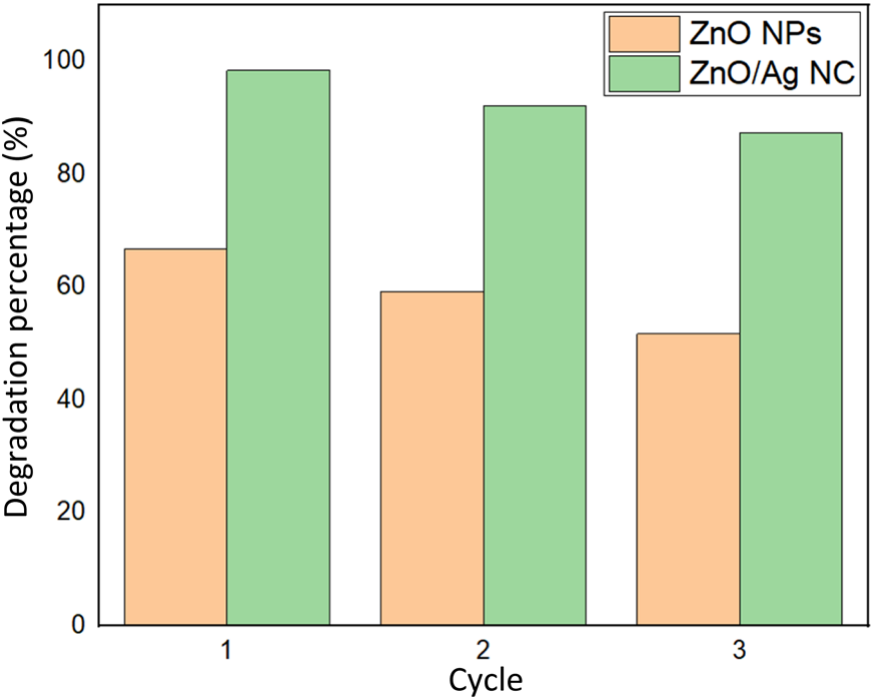
Reusability of synthesized ZnO NPs and the ZnO/Ag NC in terms of degradation percentage.

The gradual decrease in photocatalytic performance over successive cycles is typically associated with reduced availability of active sites and minor agglomeration or surface modification of the photocatalysts.^88^ Nevertheless, the ZnO/Ag NC retained a high level of activity even after three cycles, underscoring its potential for long-term photocatalytic applications in dye degradation under visible light (Fig. 8).

#### Photocatalytic degradation mechanism of the synthesized ZnO/Ag NC

**Fig. 9:**
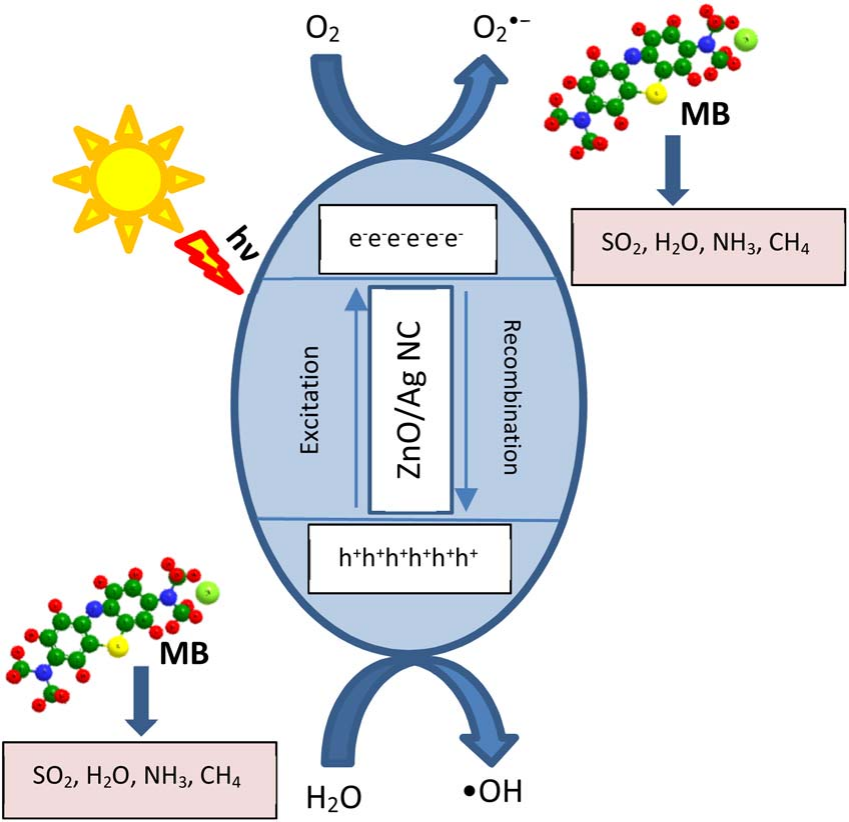
Schematic representation of the photocatalytic degradation mechanism of the ZnO/Ag NC.

The photocatalytic degradation of MB dye under illumination using the ZnO/Ag NC is primarily driven by the generation of reactive oxygen species (ROS) through the efficient separation of photogenerated charge carriers.^89^ Upon exposure to a suitable photon source, ZnO absorbs energy equal to or greater than its band gap, promoting electrons (e^−^) from the valence band (VB) to the conduction band (CB), thereby creating holes (h^+^) in the VB.^90^ The introduction of Ag NPs onto the ZnO surface significantly enhances photocatalytic performance by acting as electron sinks, facilitating charge separation and suppressing e^−^/h^+^ recombination. This mechanism is schematically represented in Fig. 9 and is described by the following reactions (eqn (30)–(36)):

30 ZnO/Ag + *hν* → e_CB_^−^ + h_VB_^+^

31 E_ZnO_^−^ → e_Ag_^−^

Ag NPs possess a lower Fermi level than ZnO, enabling the transfer of photogenerated electrons from ZnO to Ag. This Schottky junction at the ZnO/Ag interface promotes electron trapping and prolongs the lifetime of holes in the ZnO valence band.^91,92^

32 e_Ag_^−^ + O_2_ → O_2_˙^−^

33 h^+^ + H_2_O → ˙OH + H^+^

34 O_2_˙^−^ + H^+^ → HO_2_˙

35 HO_2_˙ + e^−^ → OH^−^ + ˙OH

These ROS, especially hydroxyl radicals (˙OH) and superoxide anions (O_2_^−^), are highly reactive and can non-selectively oxidize organic dye molecules.^93^

36 MB + ˙OH/O_2_˙^−^/h^+^ → intermediates → CO_2_ + H_2_O

The photocatalytic activity is further influenced by the localized surface plasmon resonance (LSPR) effect of Ag NPs, which enhances visible light absorption, extending the effective spectral range of ZnO into the visible region.^94^ This not only improves photon utilization but also assists in the continuous generation of reactive species under broader light conditions. The incorporation of Ag also introduces surface defects and oxygen vacancies in ZnO, further enhancing photocatalytic activity through improved charge mobility and increased adsorption of dye molecules.^95^ The ZnO/Ag NC thus serves as an efficient photocatalyst for the degradation of MB, showing enhanced redox potential, reduced recombination rates, and higher generation of ROS compared to ZnO NPs.

#### Antibacterial activities of ZnO NPs & the ZnO/Ag NC against *Staphylococcus aureus*, *Bacillus subtilis* & *Shigella flexneri*

**Fig. 10:**
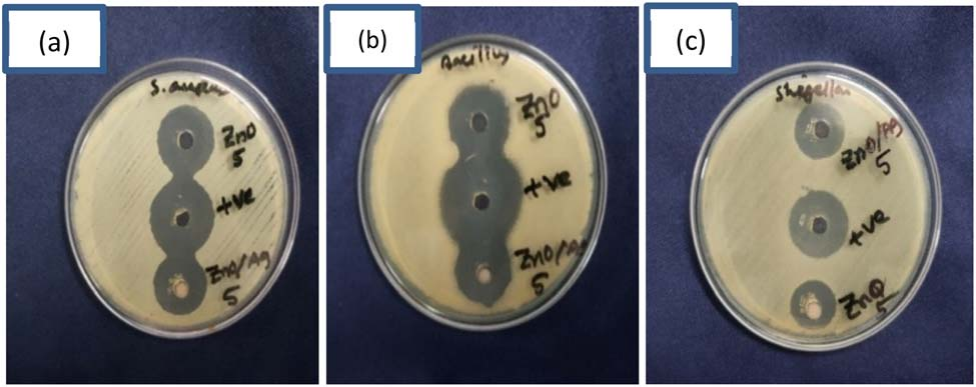
Comparison of antibacterial activity of ZnO NPs & the ZnO/Ag NC against (a) *Staphylococcus aureus*, (b) *Bacillus subtilis* & (c) *Shigella flexneri*.

The antibacterial efficacy of pure ZnO NPs and the ZnO/Ag NC was systematically evaluated against both Gram-positive (*Staphylococcus aureus*, *Bacillus subtilis*) and Gram-negative (*Shigella flexneri*) bacteria using the agar well diffusion method. The test was conducted at a concentration of 5 g L^−1^ for each sample, and the diameter of the inhibition zones around the wells was measured to assess antibacterial potency. The results, illustrated in Fig. 10 and Table 4, reveal a significant enhancement in antibacterial activity for the ZnO/Ag NC compared to pristine ZnO NPs. The ZnO/Ag NC exhibited broader inhibition zones, indicating higher antimicrobial effectiveness, especially against Gram-positive strains. The observed improvement can be attributed to the synergistic interaction between ZnO and Ag NPs. Ag incorporation enhances antibacterial activity through multiple mechanisms: (i) release of Ag^+^ ions, which disrupt bacterial cell wall integrity and interfere with protein and DNA function,^96^ (ii) increased production of reactive oxygen species (ROS), such as hydroxyl radicals and superoxide ions, which induce oxidative stress and cellular damage,^97^ and (iii) improved surface contact with bacterial membranes due to reduced particle size and increased specific surface area.^8^

**Table 4:** Diameter of the inhibition zone of the prepared samples against different microorganisms

Nanoparticle concentration	Diameter of the inhibition zone of *Staphylococcus aureus*	Diameter of the inhibition zone of *Bacillus subtilis*	Diameter of the inhibition zone of *Shigella flexneri*
ZnO (5 g L^−1^)	20	19	16
ZnO/Ag (5 g L^−1^)	20.5	20	16.5

Furthermore, while ZnO NPs themselves can generate ROS under visible light to exert bactericidal effects, the inclusion of Ag reduces charge recombination in the ZnO matrix, thereby promoting more efficient ROS formation.^98^ These radicals can damage vital cellular components like lipids, proteins, and nucleic acids, ultimately leading to bacterial cell death. The structural differences between Gram-positive and Gram-negative bacteria also influenced the sensitivity, with Gram-positive strains generally showing greater susceptibility due to their more accessible peptidoglycan layer and absence of an outer membrane barrier.^99^ Overall, the results confirm the superior broad-spectrum antibacterial potential of the ZnO/Ag NC over pure ZnO NPs, making it a promising candidate for antimicrobial applications in environmental remediation, biomedical coatings, and water purification systems.

## Conclusion

The size and lattice strain of the synthesized ZnO/Ag NC were identified as the primary causes of X-ray peak broadening. To accurately estimate crystallite size, strain-induced broadening from lattice dislocations, and energy density, a corrected Williamson–Hall (W–H) analysis was applied alongside other XRD peak broadening models. The average crystallite sizes obtained from the linear straight-line model (Scherrer's formula), W–H method, Size–Strain Plot (SSP), Halder–Wagner method, and Sahadat–Scherrer method exhibited only slight variations, confirming the consistency and reliability of the results. Among these, the Williamson–Hall method and its variants (UDM, USDM, and UDEDM) provided the most comprehensive insight into the interplay between crystallite size, lattice strain, and mechanical properties, while the SSP method offered higher accuracy for high-angle peaks. The convergence of findings across all models not only validates the robustness of the structural parameters but also demonstrates the importance of employing multiple analytical approaches for precise nanomaterial characterization. This multi-model XRD analysis framework offers a deeper understanding of the structure–property relationships in the ZnO/Ag NC and can serve as a valuable reference for optimizing nanomaterials in photocatalytic and antibacterial applications.

## Author contributions

Md. Samad Azad conceived and designed the plan. Md. Shahadat Hossain and Shassatha Paul Saikat characterized the produced samples, analyzed the data, and wrote the draft and original manuscript. Md. Rifat Hasan synthesized the sample. Shukanta Bhowmik supervised the writing and managed the required facilities.

## Conflicts of interest

There are no conflicts to declare.

## Supplementary Material

NA-007-D5NA00790A-s001

## Data Availability

The data supporting this article have been included as part of the SI. See DOI: https://doi.org/10.1039/d5na00790a.
