# Supplementary material for: Structural insights into zinc oxide–silver nanocomposite via different XRD models: rapid synthesis with photocatalytic & antibacterial applications
Source: Nanoscale Adv. 2025 Nov 3;7(24):8138–53. doi: 10.1039/d5na00790a (PMC12628709; doi:10.1039/d5na00790a)
Supplement: NA-007-D5NA00790A-s001 [file NA-007-D5NA00790A-s001.pdf]

Supplementary Information (SI) for Nanoscale Advances.
This journal is © The Royal Society of Chemistry 2025

Supplementary Information

Structural Insights into Zinc Oxide-Silver Nanocomposite via different XRD models: Rapid Synthesis with Photocatalytic & Antibacterial Applications

Md. Abdus Samad Azad [a], Md. Shahadat Hossain [b], Shassatha Paul Saikat [a], Md. Rifat Hasan [a], Shukanta Bhowmik [a*]

[a]Department of Applied Chemistry and Chemical Engineering, Noakhali Science and Technology University, Noakhali-3814, Bangladesh

[b]Department of Applied Chemistry, Noakhali Science and Technology University, Noakhali-3814, Bangladesh

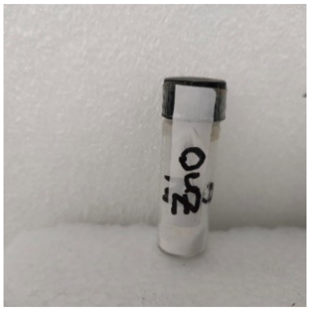

Figure S1: Synthesised ZnO nanoparticles by sol-gel method

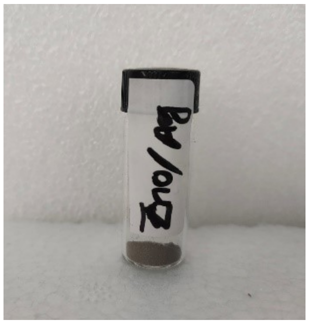

Figure S2: Synthesised ZnO/Ag NC by ultrasonic irradiation method

Photocatalytic experiments:

The photocatalytic degradation rate of MB aqueous solution was calculated by the formula:

$$\textbf{\%}\ \text{Degradation} = \frac{Co - C}{Co}\, x\ 100\% = \frac{Ao - A}{Ao}\, x\ 100\%$$

Where C_o and C are the initial and final/post-irradiation concentration of the dye, respectively; and A_o and A are the initial and final/post-irradiation absorbance of MB solution at 660nm as measured by the UV-Vis spectrophotometer, respectively.

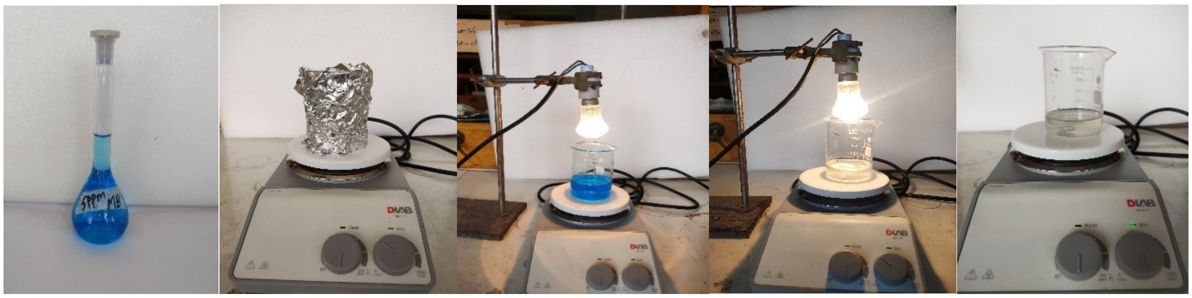

(a) (b) (c) (d) (e)

Figure S3: Photocatalytic activity of ZnO/Ag (a) 5 ppm MB solution, (b) 50 mg ZnO/Ag added in MB solution stirring 30 min in dark condition, (c) after 150 min of ZnO/Ag adding, (d) after 330 min of ZnO/Ag adding, and (e) 98.42% MB degrade by ZnO/Ag NC.

Table S1: The specific values of β (full width at half maximum) and θ (Bragg angle) used in the crystallite size calculation based on the Scherrer equation

**β °**	**θ(deg)**
0.1959	15.4775
0.1162	15.8783
0.1066	17.20505
0.1081	18.1198
0.1467	19.0512
0.2153	22.13375
0.1068	23.76425
0.1059	28.2911
0.11	31.4262
0.2332	32.20395
0.0899	33.17685
0.1125	33.9674
0.1161	34.54225
0.1122	36.2796
0.1179	38.4783

Table S2: Meaning of the symbols used in the XRD models

Symbols	Meaning
D	Crystalline size obtained from the Scherrer equation
D_L, D_c	Crystalline size obtained from the linear straight line method
K, K_a, K_b	The shape factor (0.94) used in the calculation
β_L and β_G	Lorentzian and Gaussian functions' full widths at half maximum
Xc	Crystallinity degree
δ	Dislocation density
ε	Microstrain
Y_{hkl}	Young's modulus
